# 同步推量调强放疗在局部晚期非小细胞肺癌中的应用

**DOI:** 10.3779/j.issn.1009-3419.2019.11.03

**Published:** 2019-11-20

**Authors:** 静 尤, 丹 杨, 东明 李, 蕾蕾 姜, 荣 余, 会明 于, 博 徐, 维虎 王, 安辉 石

**Affiliations:** 100142 北京，北京大学肿瘤医院暨北京市肿瘤防治研究所放疗科，恶性肿瘤发病机制及转化研究教育部重点实验室 Key Laboratory of Carcinogenesis and Translational Research (Ministry of Education/Beijing), Department of Radiation Oncology, Peking University Cancer Hospital and Institute, Beijing 100142, China

**Keywords:** 肺肿瘤, 同步推量, 有效性, Lung neoplasms, Simultaneous integrate boost, Efficacy

## Abstract

**背景与目的:**

局部晚期非小细胞肺癌（non-small cell lung cancer, NSCLC）的标准治疗方案为放疗联合化疗，但其生存仍不令人满意。随着调强放疗技术的发展，同步推量（simultaneous integrated boost, SIB）技术成为局部晚期NSCLC的研究方向。本研究拟探讨局部晚期NSCLC采用SIB调强放疗技术的有效性和安全性。

**方法:**

回顾性分析北京大学肿瘤医院2015年6月-2018年12月采用SIB技术进行放疗的局部晚期NSCLC患者资料，采用*Kaplan*-*Meier*方法进行统计分析，分析其疗效、生存及治疗相关毒性。

**结果:**

研究纳入93例患者，中位随访时间34.23个月，3年生存率、无进展生存率、无局部区域复发生存率和无远处转移生存率分别为53.0%、37.0%、50.5%和50.5%。3级放射性食管炎发生率为5.4%，≥3级放射性肺炎发生率为2.2%。

**结论:**

局部晚期NSCLC采用SIB调强放疗技术安全有效。

## Abstract (en)

**Background and Objective:**

The standard treatment for locally advanced non-small cell lung cancer (NSCLC) is radiotherapy concurrent with chemotherapy, but the survival was not satisfied. With the development of intensity modulated radiotherapy, simultaneous integrated boost technique (SIB) becomes the research direction of locally advanced NSCLC. The aim of this study is to investigate the efficacy and safety of SIB intensity modulated radiotherapy technique for locally advanced NSCLC.

**Methods:**

We retrospectively reviewed the clinical data of locally advanced NSCLC who were treated with radiotherapy by SIB technique in Peking University Cancer Hospital from June 2015 to December 2018. *Kaplan*-*Meier* method was used for analysis.

**Results:**

Ninty-three patients were included in the analysis. After a median follow-up of 34.23 months, 3-year overall survival (OS), progression-free survival (PFS), local-recurrence free survival (LRFS) and metastasis free survival (MFS) rates were 53.0%, 37.0%, 50.5% and 50.5%, respectively. The incidence of grade ≥3 esophagitis was 5.4%. There were 2 (2.2%) patients experiencing grade ≥3 radiation-related pneumonia.

**Conclusion:**

Radiation with SIB intensity modulated radiotherapy technique is effective and safe for patients with locally advanced NSCLC.

肺癌是我国最常见的恶性肿瘤之一，也是全球肿瘤死亡最主要的原因^[1]^。其中，非小细胞肺癌（non-small cell lung cancer, NSCLC）约占肺癌的85%，30%发现时已处于局部晚期，以放化疗为主的综合治疗成为局部晚期NSCLC的标准治疗^[2-4]^。美国国立综合癌症网络（National Comprehensive Cancer Network, NCCN）指南推荐，NSCLC根治性放疗剂量为60 Gy-70 Gy。随着放疗技术的发展，调强放疗技术由于其可提高肿瘤控制率、降低肺毒性和心脏毒性逐渐成为常用的放疗技术^[5]^，但计划大体肿瘤体积（planning gross tumor volume, PGTV）和计划靶体积（planning target volume, PTV）通常给予相同的放疗剂量。RTOG 0617研究将放疗剂量由60 Gy提高至74 Gy，并没有带来生存获益，反而增加了治疗相关毒性^[6]^。随着调强放疗技术的实现，研究者开始尝试采用同步推量（simultaneous intergrate boost, SIB）技术。SIB技术是指在同一个射野内，对PGTV和PTV给予不同的剂量，PTV分次剂量为1.8 Gy-2.0 Gy，而PGTV分次剂量略高，为2.12 Gy-2.5 Gy。采用这种技术能使肿瘤病灶得到较高放疗剂量的同时，而不增加靶区周围正常组织的剂量^[7, 8]^，从而达到提高肺癌局部控制率的同时不增加周围正常组织毒性的目的，也能达到缩短总治疗时间的目的^[8, 9]^。本研究拟通过分析SIB调强放疗技术在局部晚期NSCLC中的有效性和安全性，为局部晚期NSCLC寻找更适合的放疗剂量和方案提供更多的依据。

## 1 材料与方法

### 1.1 一般资料

回顾性分析2015年6月-2018年12月于北京大学肿瘤医院接受SIB技术进行放疗的局部晚期NSCLC患者的临床资料。纳入标准：①病理类型为NSCLC；②临床分期为局部晚期；③除外远处转移；④东部肿瘤协作组（Eastern Cooperative Oncology Group, ECOG）评分0分-1分；⑤采用SIB调强放疗技术进行根治性放疗；⑥无严重心肺功能疾病；⑦血常规及肝肾功能正常。排除标准：①有远处转移；②放疗未采用SIB技术。剔除标准：①数据记录质量差，资料不完整；②患者因其他因素拒绝治疗。

### 1.2 化疗方案

患者化疗方案采用以顺铂为主的两药联合化疗方案。

### 1.3 放疗方案

所有患者采用胸部增强计算机断层扫描（computed tomography, CT）进行定位扫描，扫描层厚5 mm，扫描范围为下颌骨下缘至肝下缘。CT图像通过网络传送至治疗计划系统工作站。根据ICRU 62号文件的定义进行靶区勾画，大体肿瘤体积（gross tumor volume, GTV）定义为影像学可见的原发灶以及阳性淋巴结。原发灶临床靶体积（clinical target volume, CTV）为原发灶GTV三维外扩，鳞癌三维外扩6 mm，腺癌三维外扩8 mm，再根据解剖边界进行调整，淋巴结CTV参考化疗前的阳性淋巴结，为相应阳性淋巴结引流区。CTV基础上三维外扩5 mm形成PTV，GTV基础上三维外扩5 mm形成PGTV。PTV总剂量为60 Gy（单次剂量2.0 Gy），对PGTV采用SIB技术，总剂量为66 Gy-70 Gy（单次剂量2.2 Gy-2.3 Gy）。危及器官限量：双肺V5≤65%，双肺V10≤50%，双肺V20≤30%，双肺平均剂量≤20 Gy；心脏V40≤30%，V30≤40%，脊髓Dmax为45 Gy。采用每日一次放疗，周一至周五，每周5日。放疗采用瓦里安直线加速器，6 MV-10 MV X线进行。放疗开始前3 d及每周行锥形束CT验证患者位置。

### 1.4 疗效、毒性评价及随访

根据实体瘤疗效评价标准（Response Evaluation Criteria In Solid Tumors, RECIST）1.1标准^[10]^进行评效。治疗期间不良反应每周评价一次，根据美国国立癌症研究所（National Cancer Institute, NCI）常见毒性反应标准（Common Terminology Criteria for Adverse Events, CTCAE）版本4.0^[11]^进行评价。治疗结束第1个月，之后2年内每3个月，第3-5年每6个月，5年后每12个月随访一次。随访内容包括患者症状、查体、血常规、生化、肿瘤标志物及影像学检查。

### 1.5 统计学方法

所有数据资料采用SPSS 21.0软件分析。总生存（overall survival, OS）定义为从诊断日期开始至任何原因所致死亡的日期或末次随访日期。无进展生存（progression-free survival, PFS）定义为从诊断日期开始至疾病进展、死亡的日期或末次随访日期。局部区域无进展生存（local recurrence free survival, LRFS）定义为从诊断日期开始至局部失败的日期或末次随访日期。无远处转移生存（metastasis free survival, MFS）定义为从诊断日期开始至出现远处转移的日期或末次随访日期。采用*Kaplan*-*Meier*法计算分析患者的生存情况。

## 2 结果

### 2.1 患者临床特点

2015年6月-2018年12月期间在北京大学肿瘤医院行胸部放疗的肺癌患者共2, 946例，其中接受根治性放疗的局部晚期肺癌患者共415例。其中，筛选出采用SIB技术调强放疗的93例局部晚期NSCLC患者进行分析。患者临床资料具体见表 1。患者中位年龄为62岁（范围34岁-80岁）。其中，2例为IIb期，31例为Ⅲa期，47例为Ⅲb期，13例为Ⅲc期。53例为鳞癌，33例为腺癌，7例为其他病理类型。65例接受同步放化疗，28例接受序贯放化疗。

**1:** 非小细胞肺癌患者采用同步推量技术进行放疗的临床资料 Patient characteristics with non-small cell lung cancer by simultaneous integrate boost technique

Clinical characteristics	*n*	Percentage (%)
Gender		
Male	72	77.4
Female	21	22.6
Age (yr)		
Median (range)	62.0 (34-80)	-
≤60	40	43.0
> 60	53	57.0
History of smoking		
Yes	23	37.7
No	38	62.3
Primary pulmonary diseases		
Yes	5	5.4
N0	88	94.6
T stage		
T1	8	8.6
T2	31	33.3
T3	21	22.6
T4	33	35.5
N stage		
N0	4	4.3
N1	5	5.4
N2	54	58.1
N3	30	32.3
AJCC staging (8^th^ edition)		
Ⅱb	2	2.2
Ⅲa	31	33.3
Ⅲb	47	50.5
Ⅲc	13	14.0
Pathologic pattern		
Squamous cell carcinoma	53	57.0
Adenocarcinoma	33	35.5
Others	7	7.5
Chemoradiation regimen		
Concurrent chemoradiation	65	69.9
Sequential chemoradiation	28	70.1
PGTV volume (mL)		
Median (minimum-maximum)	158.9 (28.1-926.3)	-
PTV volume (mL)		
Median (minimum-maximum)	358.5 (45.5-1, 045.6)	-
AJCC: American Joint Committee on Cancer; PGTV: planning gross tumor volume; PTV: planning target volume.		

### 2.2 危及器官限量

双肺、食管及心脏的危及器官限量见表 2。

**2:** 采用同步推量技术进行放疗的非小细胞肺癌患者的危及器官限量 （Median±SD） Organs at risk for non-small cell lung cancer by simultaneous integrate boost technique  (Median±SD)

Organs at risk	Data
Lung	
V20 (%)	23.30±5.91
V10 (%)	36.20±9.26
V5 (%)	50.84±10.82
MLD (cGy)	1, 376.15±281.75
Esophagus	
Dmean (cGy)	2, 458.70±1, 292.45
Dmax (cGy)	6, 869.70±1, 377.05
Heart	
V40 (%)	11.70±14.10
V45 (%)	10.40±11.75
Dmax (cGy)	1, 242.50±1, 022.55

### 2.3 近期疗效及毒副反应

#### 2.3.1 近期疗效

93例患者中，9例（9.7%）达到完全缓解，54例（58.1%）达到部分缓解，25例（26.9%）患者病情稳定，5例（5.4%）患者病情进展。总客观有效率（完全缓解及部分缓解率）为67.7%（63/93）。

#### 2.3.2 放疗相关毒性

2级及以上放射性食管炎发生率为46.3 %（43/93），其中有5例（5.4 %）患者出现3级放射性食管炎。放射性肺炎发生率为22.6 %（21/93），其中≥3级放射性肺炎发生率为2.2 %（2/93）（表 3）。

**3:** 采用同步推量放疗的非小细胞肺癌患者的放疗相关毒性 [*n* (%)] Treatment-related toxicities for patients with non-small cell lung cancer by simultaneous integrate boost technique  [*n* (%)]

Treatment-related toxicities	Grade 0	Grade 1	Grade 2	Grade 3	Grade 4	Grade 5
Leukemia	34 (36.6)	21 (22.6)	28 (30.1)	8 (8.6)	2 (2.2)	0 (0.0)
Neutropenia	56 (60.2)	14 (15.1)	18 (19.4)	4 (4.3)	1 (1.1)	0 (0.0)
Anemia	63 (67.7)	24 (25.8)	5 (5.4)	1 (1.1)	0 (0.0)	0 (0.0)
Thrombocytopenia	78 (83.9)	12 (12.9)	2 (2.2)	1 (1.1)	0 (0.0)	0 (0.0)
Esophagitis	30 (32.3)	20 (21.5)	38 (40.9)	5 (5.4)	0 (0.0)	0 (0.0)
Pneumonitis	72 (77.4)	13 (14.0)	6 (6.5)	0 (0.0)	0 (0.0)	2 (2.2)
Nausea	61 (65.6)	25 (26.9)	5 (5.4)	0 (0.0)	0 (0.0)	0 (0.0)

### 2.4 首次复发或进展模式

中位随访时间34.23个月，截止至末次随访时间，有51例（54.8%）患者存活，56例（60.2%）患者出现肿瘤进展。其中，有19例患者出现单纯局部复发或进展，21例出现单纯远处转移，13例出现局部复发/进展及远处转移。

### 2.5 生存情况

中位随访时间34.23个月（3.13个月-71.2个月），共42例患者死亡，有33例患者死于疾病进展，2例死于放射性肺炎，1例死于咯血，2例死于心血管事件，1例死于呼吸衰竭，3例患者死亡原因不详。患者1年、2年和3年OS率分别为87.0%、64.0%和53.0%，1年、2年和3年PFS率分别为66.0%、43.0%和37.0%，1年、2年和3年LRFS率分别为81.0%、63.0%和50.5%，1年、2年和3年MFS率分别为78.0%、65.0%和50.5%。生存结果见图 1。

**1:**
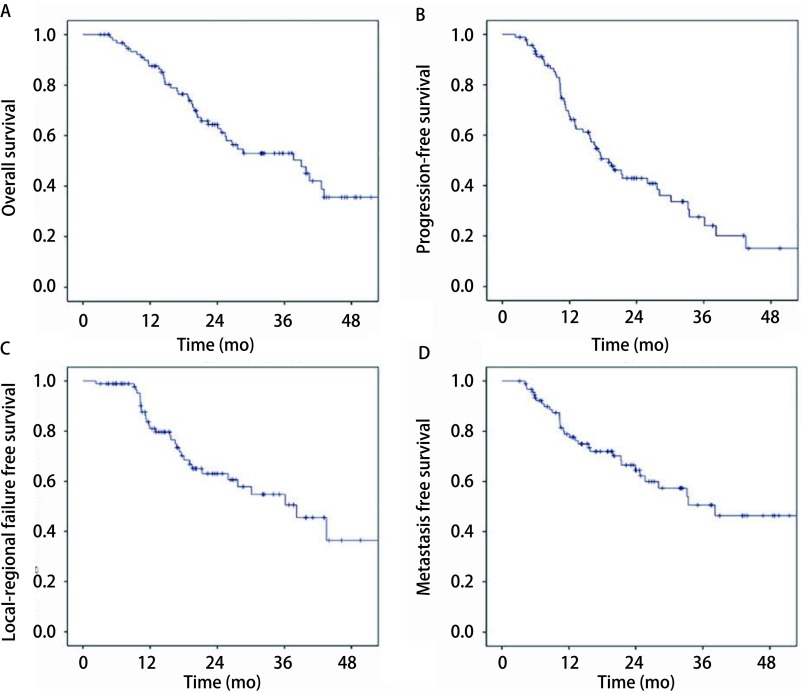
非小细胞肺癌患者行同步推量放疗的生存曲线。A：总生存；B：无进展生存；C：局部区域无复发生存；D：无远处转移生存。 Survival curve of the patients with non-small cell lung cancer by simultaneous integrate boost technique. A: Overall survival; B: Progression-free survival; C: Local-regional failure free survival; D: Metastasis free survival.

## 3 讨论

局部晚期NSCLC目前较常采用的放疗方案为常规分割放疗，PGTV和PTV采用相同的放疗剂量，但以常规放疗为主的综合治疗的疗效并不令人满意。治疗失败的原因主要包括局部未控、复发和远处转移^[12, 13]^。剂量学方面，有研究^[14]^显示，与常规分割放疗技术相比，局部晚期NSCLC采用SIB调强放疗技术可提高肿瘤区域剂量14.7 Gy（22%），且未明显增加周围正常组织受照剂量。本研究中，SIB技术进一步提高了肿瘤的有效剂量，生物有效剂量（biological effective dose, BED）达到80.5 Gy-86.1 Gy，而常规分割放疗的BED为72.0 Gy。

目前，有几项研究探讨了SIB技术在NSCLC中应用的可行性。RTOG 8407研究^[15]^中PTV每日放疗剂量为1.8 Gy，同时对PGTV采用剂量递增方式进行SIB，结果显示，当PGTV剂量从63 Gy提高至70.2 Gy，2年生存率由16%提升至21%，且治疗相关急性和晚期毒性并没有显著增加。Sun等^[16]^纳入97例NSCLC患者进行SIB放疗（43例）或常规分割放疗（54例），SIB放疗组PTV剂量为46.8 Gy（单次剂量1.8 Gy），将PGTV推量至65 Gy（单次剂量2.5 Gy），而常规分割放疗组接受70.8 Gy（单次剂量1.9 Gy）放疗，结果显示，SIB放疗有效率较常规分割放疗提高了21.7%（69.8% *vs* 48.1%），且未增加≥3级非血液学毒性。Izmirli等^[17]^的一项前瞻性Ⅱ期研究纳入30例Ⅲ期NSCLC患者，在接受27 Gy放疗后开始行SIB技术放疗，将PGTV推量至63 Gy，结果与上述报道相似。Fondevilla等^[18]^对64例行SIB技术进行放疗的局部晚期NSCLC患者进行分析，PTV剂量为56 Gy，将PGTV推量至68 Gy，1年和2年OS率分别为79%和46%。而本研究采用SIB调强放疗技术对局部晚期NSCLC进行放疗，维持PTV总剂量60 Gy不变，将PGTV总剂量推量至66 Gy-70 Gy（单次剂量2.2 Gy-2.3 Gy），3年OS率为53.0%，3年LRFS率为50.5%。也有一些研究^[19]^对亚临床病灶给予更低的处方剂量（中位50.4 Gy），与常规分割放疗60 Gy组相比，3年LRFS率并没有增加（53% *vs* 45%, *P*=0.39）。以上数据提示，对局部晚期NSCLC采用SIB调强放疗技术可取得较好的疗效。

采用SIB调强放疗技术在增加肿瘤放疗剂量的同时未显著增加亚临床区域的放疗剂量，因而理论上不会显著增加危及器官的限量。Xia等^[20]^的研究从物理学的角度显示，采用SIB技术可以达到更低的危及器官限量。有研究^[21]^显示，V20是预测急性放射性肺炎的重要指标，V20与≥2级放射性肺炎发生率之间显著相关（*P*=0.001）。本研究中双肺V20中位值为23.3%，低于常规V20要求，≥2级放射性肺炎发生率为8.7%。而Wang等^[19]^的研究中双肺V20的中位值为25.26%，略高于本研究，可能由于本研究PTV体积略小有关（PTV中位值分别为358.5 mL *vs* 504 mL）。其他一些采用SIB技术进行放疗的研究均显示出放射性肺炎并没有随着调强放疗技术的应用而增加^[22, 23]^。放射性食管炎方面，Mustafa等^[17]^的研究中1级-2级放射性食管炎发生率为85%，韩国Cho等^[24]^的Ⅱ期研究中≥2级放射性食管炎发生率为59.2%。本研究中放射性食管炎发生率与以上研究结果相似。因而，局部晚期NSCLC采用SIB调强放疗技术耐受性良好。

RTOG 0617研究结果^[6]^显示，由于同时提高肿瘤病灶和亚临床病灶的剂量并没有带来生存的获益，反而增加了治疗相关毒性。近些年来，越来越多的研究通过各种技术或手段使SIB调强放疗技术成为可研究的方向。一项Ⅱ期研究^[25]^基于正电子发射型计算机断层显像（positron emission computed tomography, PET）/CT引导的SIB调强放疗技术在NSCLC放疗中取得了较好的局部区域控制率。另一项Ⅰ期研究^[26]^采用质子放疗技术进行SIB，可将NSCLC放疗的剂量安全提升至72 Gy。因此，随着放疗技术的不断进步，为了进一步提高局部晚期NSCLC的局部控制和生存率，SIB调强放疗技术成为安全可行的一种手段。

综上所述，本研究显示，SIB调强放疗技术在NSCLC放疗中可以作为一种较安全和有效的治疗手段。但本研究为回顾性研究，样本量较小，未来需要大样本前瞻性研究探讨SIB调强放疗技术的可行性以及探讨如何更好地利用更先进的放疗技术和手段。
